# Mutational Landscape for Indian Hereditary Breast and Ovarian Cancer Cohort Suggests Need for Identifying Population Specific Genes and Biomarkers for Screening

**DOI:** 10.3389/fonc.2020.568786

**Published:** 2021-01-21

**Authors:** Mohammed Shaad N. Kadri, Komal M. Patel, Poonam A. Bhargava, Franky D. Shah, Nutan V. Badgujar, Bhoomi V. Tarapara, Prabhudas S. Patel, Mohammed Inayatullah Shaikh, Krati Shah, Apurva Patel, Shashank Pandya, Hemangini Vora, Chaitanya G. Joshi, Madhvi N. Joshi

**Affiliations:** ^1^ Gujarat Biotechnology Research Centre, Department of Science and Technology, Government of Gujarat, Gandhinagar, India; ^2^ Gujarat Cancer Research Institute, Civil Hospital, Ahmedabad, India; ^3^ Clinical Genetics, ONE-Centre for Rheumatology and Genetics, Vadodara, India

**Keywords:** genetic testing, next generation sequencing, amplicon sequencing, non-BRCA genes, customized multi-gene panel, hereditary breast and ovarian cancer, BRCA1 and BRCA2

## Abstract

**Background:**

Breast and ovarian cancers are the most prevalent cancers and one of the leading causes of death in Indian women. The healthcare burden of breast and ovarian cancers and the rise in mortality rate are worrying and stress the need for early detection and treatment.

**Methods:**

We performed amplicon sequencing of 144 cases who had breast/ovarian cancer disease (total 137 cases are patients and seven are tested for BRCA1/2 carrier) Using our custom designed gene panel consisting of 14 genes, that are associated with high to moderate risk of breast and ovarian cancers. Variants were called using Torrent Variant Caller and were annotated using ThermoFisher’s Ion Reporter software. Classification of variants and their clinical significance were identified by searching the variants against ClinVar database.

**Results:**

From a total of 144 cases, we were able to detect 42 pathogenic mutations in [40/144] cases. Majority of pathogenic mutations (30/41) were detected in BRCA1 gene, while (7/41) pathogenic mutations were detected in BRCA2 gene, whereas, (2/41) pathogenic mutations were detected in TP53 gene and BRIP1, PALB2, and ATM genes respectively. So, BRCA genes contributed 88.09% of pathogenic mutations, whereas non-BRCA genes contributed 11.91% of pathogenic mutations. We were also able to detect 25 VUS which were predicted to be damaging by *in silico* prediction tools.

**Conclusion:**

Early detection of cancers in the Indian population can be done by genetic screening using customized multi-gene panels. Indications of our findings show that in the Indian population, apart from the common BRCA genes, there are other genes that are also responsible for the disease. High frequency mutations detected in the study and variants of uncertain significance predicted to be damaging by *in silico* pathogenicity prediction tools can be potential biomarkers of hereditary breast and ovarian cancer in Indian HBOC patients.

## Introduction

Breast and ovarian cancer are the most prevalent cancer and leading cause of death in Indian women. The incidence of breast cancer cases in India in 2018 was estimated to be 162,468, which is 27.6% of all cancer cases in females. Whereas, for ovarian cancers, it was estimated to be 36,170, which is 6.2% of all cancer cases in females (1). Gujarat ranks first in deaths due to breast cancer and seventh in deaths due to ovarian cancer among other states of India (2). Moreover, the ratio of number of deaths to new cases in case of breast cancer is increased to 56.30% (87090/162648) in India which is much higher as compared to F.6% (131,347/458,718) in Europe and 19% (48,850/256,222) in the US (1, 3, 4). In case of ovarian cancer burden in India, the ratio of number of deaths to new cases is 66.39% (24015/36170) which is also higher in comparison to Europe 65.77% (44576/67771) and US 57.24% (14008/24469) (1, 3, 4).

Hereditary breast and ovarian cancer (HBOC) is an inherited disorder in which the risk of breast and ovarian cancers is higher than normal BOC. About 5–10% of breast cancers and 10–15% of ovarian cancers can be attributed to HBOC (5). HBOC is characterized by bilateral cancer with a family history of breast or ovarian cancer in relatives. BRCA1 and BRCA2 (BRCA1/2) genes are maximally associated with predisposition to HBOC; however, in addition to the BRCA1/2 genes, the National Comprehensive Cancer Network (NCCN) guidelines have been expanded to incorporate non-BRCA genes into gene panels for increased medical management (6). According to the 2014 NCCN guidelines for multi-gene testing, women with mutations in the non-BRCA genes, such as TP53, PTEN, CDH1, ATM, CHEK2, or PALB2 tumor suppressor genes and others also have an increased risk of breast and ovarian cancer (2, 6). In one of the studies, in individuals who underwent multigene testing, the researchers found that 3.8% of BRCA1/2 mutation-negative individuals harbored deleterious mutations in other hereditary cancer predisposition genes. 40–50% more individuals with mutations in these cancer susceptibility genes could be identified by multigene panel testing as compared to testing for BRCA1 and BRCA2 genes only (7). A high rate of mortality in India due to cancers is mainly because of late diagnosis and delayed treatment (8). Thus, it is suggested that non-BRCA genes must also be tested so that deleterious non-BRCA mutations can also be covered and presentation of HBOC related mutations in Indian population can be achieved. This would enable us to gather clinically useful data for HBOC risk assessment and diagnosis in Indian population. For this, gene panels that cover both BRCA1/2 and non-BRCA genes should be incorporated so that a detailed picture of mutations contributed by both BRCA and non-BRCA genes can be obtained (2, 9–12). In our study we used 14 gene panels, in accordance with 2014 NCCN guidelines and other literature, for determining the contribution of BRCA and non-BRCA genes in HBOC in the Indian population (6, 13). We also sought to find out mutations specific/prevalent to the Indian cohort, so that they can be used as hotspots for readily detecting HBOC in India. For this, we used next generation sequencing (amplicon sequencing) based approach to screen 14 high to moderate risk genes associated with breast and ovarian cancers in a cohort of 144 Indians, related and unrelated individuals, with at least a 1^st^ degree family history of breast and/or ovarian cancer (6).

The percentage of detection of pathogenic mutations in non-BRCA genes in patients with breast and ovarian cancers is 17.64%, which indicates that, testing of non-BRCA genes in combination of BRCA1/2 genes is equally necessary for a complete risk assessment and for developing a more accurate method for early detection, diagnosis and treatment (14, 15).

## Materials and Methods

### Patient Selection

**Figure 1:**
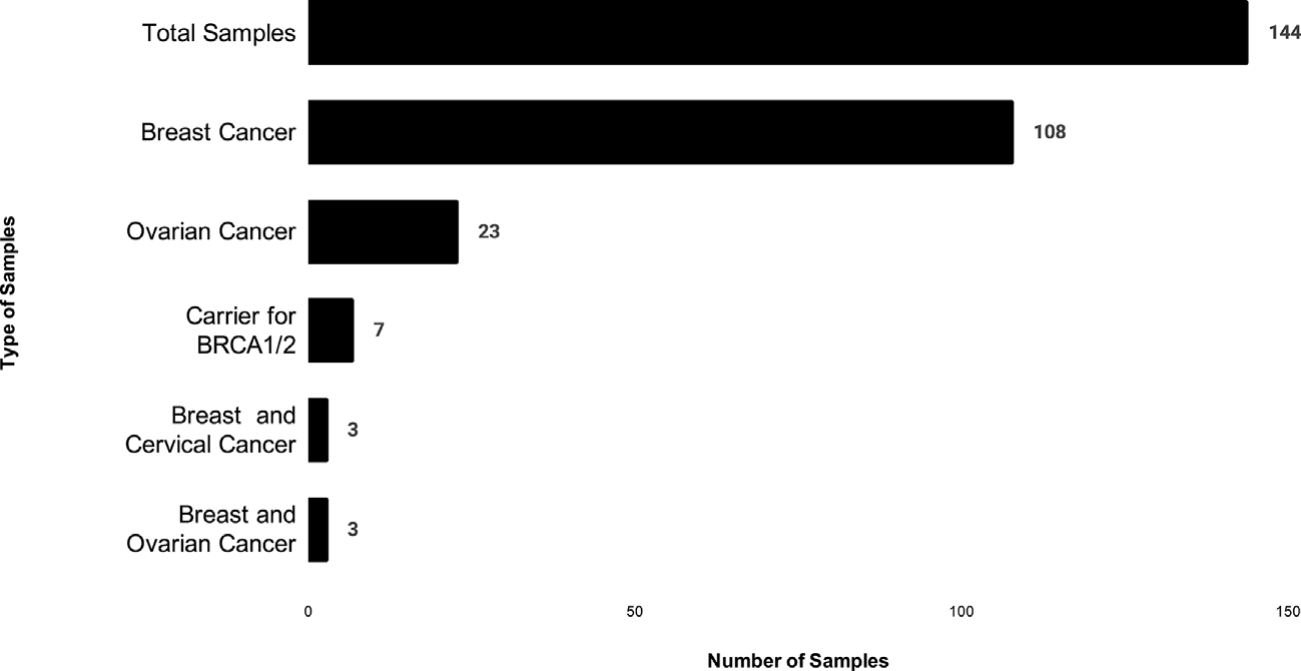
The distribution of patients based on cancer type in which X-axis shows number of patients, and Y- axis shows type of cancer in our study.

**Figure 2:**
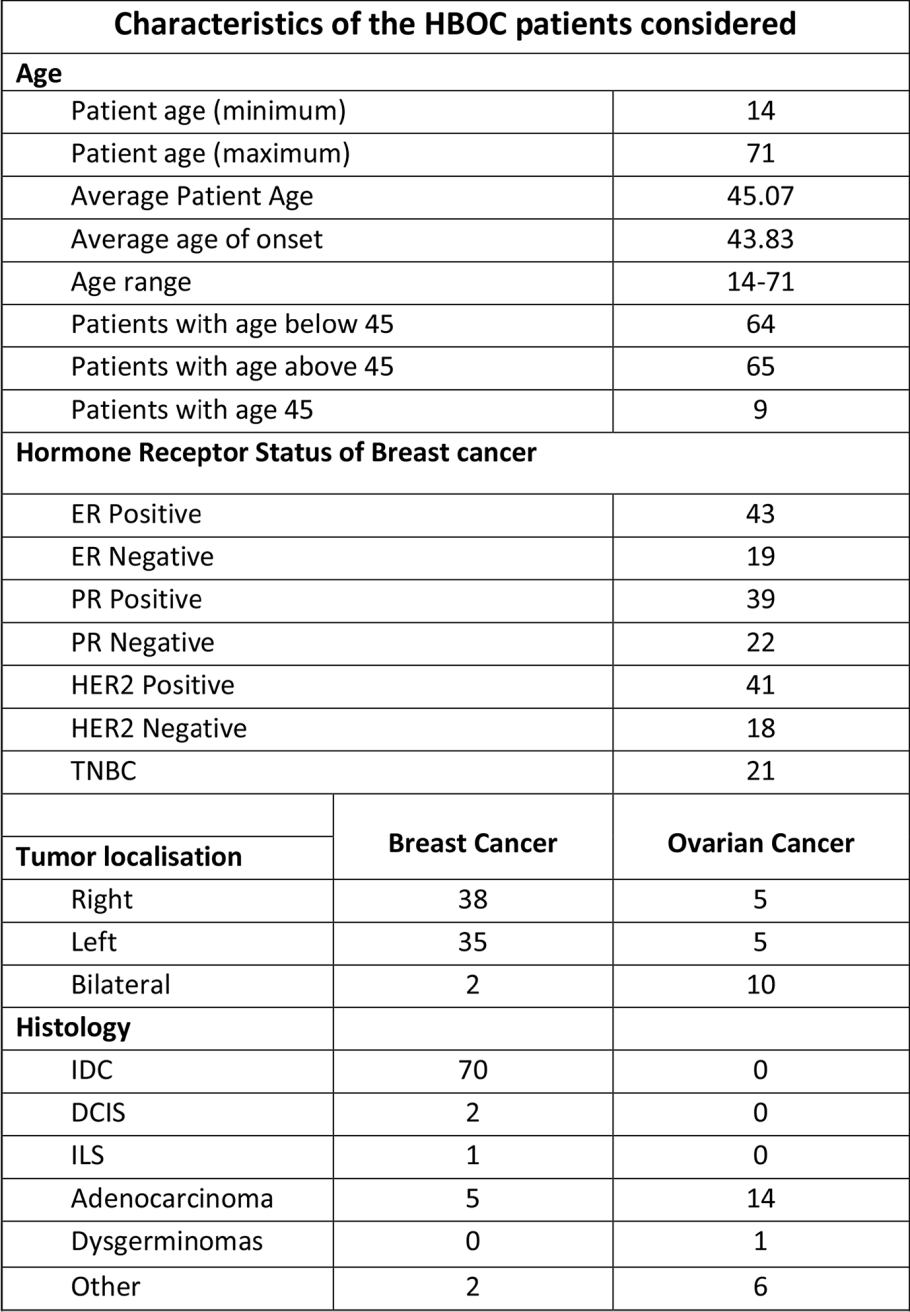
Characteristics of HBOC patients considered for the study including patients’ age, tumor localization and histology.

The study was conducted and approved in accordance with the Gujarat Cancer Research Institute’s Institutional Review Board Committee at GCRI, Civil Hospital, Ahmedabad, Gujarat, India. Patients undergoing treatment or treated earlier and having a family history of breast or ovary cancer in the first or second degree relative were selected for the study. Inclusion of patients was made according to the ICMR guidelines (16). Written informed consent was also obtained from each participant of the study. Clinical and pathologic details were retrieved from the medical records. Genetic counseling of breast and ovary cancer patients was performed prior to patient’s selection, through cancer camps held at Gujarat Cancer and Research Institute. Out of 144 cases, 108 patients were having breast cancer, and 23 patients were having ovarian cancer, three patients were having breast and cervical cancer whereas three patients had breast and ovarian cancer. Seven carrier cases were enrolled as relatives of two different patients **Figure 1**. Majority of the patients selected (64/144) were below 45 years with a mean age of 45 years at the time of diagnosis **Figure 2**. Out of 144 cases, 18 cases were from six different families; hence they were interrelated, and 126 cases were unrelated individuals from singular families.

### Amplicon Sequencing

#### Customized Multi-Gene Panel

In this study, a custom Ion AmpliSeq™ Panel of 14 HBOC associated high-penetrance and moderate risk genes as mentioned in the NCCN guidelines and other literature was considered for mutational profiling of Indian HBOC patients (6, 13). The 14 HBOC genes, BRCA1 (NM_007300.3), BRCA2 (NM_000059.3), TP53 (NM_000546.4), PTEN (NM_000314.4), CDH1 (NM_004360.3), STK11 (NM_000455.4), ATM (NM_000051.3), BARD1 (NM_000465.3), BRIP1 (NM_032043.2), CHEK2 (NM_007194.3), ERBB2 (NM_004448.3), NBN (NM_002485.4), PALB2 (NM_024675.3), RAD51C (NM_058216.1), were sequenced using 965 amplicons in two pools.

#### Sample Preparation

Genomic DNA was extracted from blood samples using QIAamp DNA Blood Mini kit (QIAGEN, Germany). The concentration of DNA was determined using Qubit Fluorometer 2.0 and 4.0^®^(ThermoFisher), and purity of DNA was determined using QIAxpert (QIAGEN).

#### Sequencing

Multiplex PCR was performed using 50–100 ng genomic DNA with a premixed primer pool and Ion AmpliSeq™ HiFi master mix (Ion AmpliSeq™ Library Kit 2.0) for 2 min at 99°C, followed by 16 cycles of 99°C for 15 s and 60°C for 4 min, ending with a holding period at 10°C. The PCR amplicons were treated with 2 µl FuPa reagent to partially digest primer sequences and phosphorylate the amplicons at 50°C for 10 min, followed by 55°C for 10 min, then 60°C for 20 min. The amplicons were ligated to adapters with the diluted barcodes of the Ion Xpress™ Barcode Adapters kit (ThermoFisher) for 30 min at 22°C then 68°C for 5 min followed by 72°C for 5 min. Adaptor ligated amplicon libraries were purified using Agencourt^®^ AMPure^®^ XP reagents (Beckman Coulter, Tokyo, Japan). The library concentration was determined using Qubit dsDNA HS Assay Kit (Thermo Fisher Scientific), then each library was diluted to 100 pmol, and the same amount of libraries was pooled for one sequencing reaction. Next, emulsion PCR was carried out using the Ion OneTouch™ 2 System (ThermoFisher) according to the manufacturer’s instructions. Template-positive Ion Sphere™ Particles were then enriched with Dynabeads^®^ MyOne™ Streptavidin C1 Beads (ThermoFisher) using an Ion OneTouch™ ES system (ThermoFisher). Massively parallel sequencing was carried out on Ion Proton and Ion S5™ using the Ion Proton Sequencing 200 Kit and Ion S5 540 and Ion S5 520 and 530 OT2 and sequencing kit according to the manufacturer’s instructions.

### Data Processing

The raw sequence data was processed using standard Ion Torrent Suite™ Software running on the Torrent Server. Raw signal data were analyzed using Torrent Suite™. The pipeline includes signal processing, base calling, quality score assignment, adapter trimming, PCR duplicate removal, read alignment to human genome 19 reference (hg19), quality control of mapping quality, coverage analysis **Figure 2**. Identification of sequence variants was carried out *via* Torrent Variant Caller Plugin software (ThermoFisher), and coverage of each amplicon was obtained by the Coverage Analysis Plugin software (ThermoFisher). The variant call parameter setting was germline high stringency. Mutations with low quality, occurring intronic regions were discarded from the datasets. Following this, annotation of the remaining high quality single-nucleotide variants, insertions, deletions, and splice site alterations was performed using Ion Reporter™ Server System (ThermoFisher). Mutations with known clinical significance were identified by searching variants against ClinVar database. Functional consequences of variants which are novel or which were under the category of variants of uncertain significance or those which could not be classified as pathogenic or likely pathogenic by prior reports were assessed using 17 *in silico* pathogenicity prediction tools which were incorporated in the search engine named Varsome: The Human Genomics Community (17).

### Variant Classification

Classification of variants was done according to the American College of Medical Genetics and Genomics (ACMG) recommendations for standard interpretation and reporting of sequence variations (18). The variants were classified into five categories, such as pathogenic, likely pathogenic, variant of uncertain significance (VUS), likely benign, and benign.

### Mutational Pattern of Indian Cohort Assessment

In order to check the differences in mutational pattern of Indian HBOC patients with that of other populations, we compared the minor allele frequencies of clinically significant mutations from the Indian HBOC patients with that of South Asian, European (Finnish and non-Finnish), Ashkenazi, Latino, East Asian, and African populations using the GnomAD browser (19).

## Results

Amplicon sequencing targeting 14 genes in 144 samples generated 42.76 GB of sequences with 428 million high quality sequencing reads. Mean sequencing depth of high quality reads was 3,602×, and >95% reads were mapped on to the target regions. Alignment of sequencing reads was done on to the targeted regions of reference genome hg19 which produced a total of 7,626 SNVs and INDELs in 144 samples, of which 3,488 variants were observed to be occurring in exonic region, 3,936 in intronic region, 11 in splice sites, and 191 in untranslated regions. Introns, synonymous variants, and variants below 100× coverage were removed to finally obtain 1,770 variants.

### Distribution of Variants in Patients Across 14 Genes

**Figure 3:**
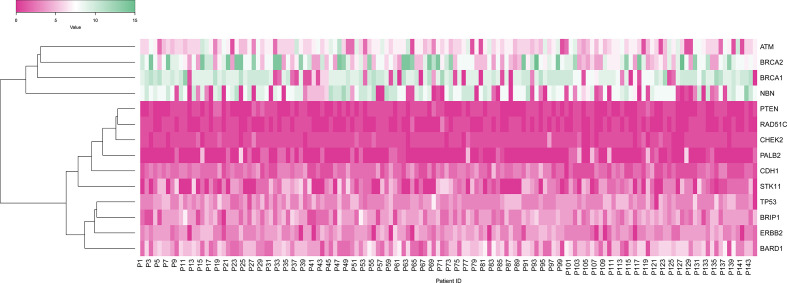
Heat map showing gene-wise distribution of variants in 144 patients in which X-axis shows patients, and Y-axis shows the gene variants.

To start with, we constructed a heat map to obtain the distribution of Variants among all 144 cases across all 14 genes included in the multi-gene panel. Mutations were detected in all 14 genes tested: ATM, BARD1, BRCA1, BRCA2, BRIP1, CDH1, CHEK2, EBB2, NBN, PALB2, PTEN, RAD51C, STK11, TP53. Distribution of variants revealed that the majority 30.50% was in the BRCA1/2 genes as compared to the 12 non-BRCA genes altogether 69.5%. Of the BRCA mutations, the majority 14.96% were detected in BRCA1 and 15.53% of mutations were detected in BRCA2. In the non-BRCA genes, the mutation detection rate was as follows: NBN [12.20%], ATM (11.36%), BARD1 (10.11%), ERBB2 (8.14%), BRIP1 (6.81%), TP53 (6.74%), STK11 (4.31%), CDH1 (4.06%), CHEK2 (1.84%), PALB2 (1.23%), PTEN (1.04%) and RAD51C [1.57%] **Figure 3**.

### Categorization of All Mutations Into ClinVar Categories

After filtration, 1,770 variants obtained were classified based on searching each variant against the ClinVar database, and according to their clinical significance, the variants were categorized into different categories, such as, Pathogenic variants, Likely Pathogenic variants, Variants of Uncertain Significance and Benign/Likely benign variants. 2.37% variants were placed into the category of Pathogenic variants, 1.41% variants were placed into the category of variants of uncertain significance (VUS), 96.29% variants were placed into the category of Benign/Likely benign variants.

#### Pathogenic Mutations

**Figure 4:**
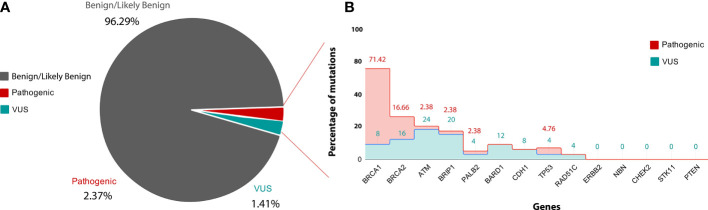
**(A)** is showing classification of variants into clinvar categories: Benign/likely Benign (Grey), VUS (blue) and pathogenic (red) according to ACMG guideline. **(B)** is showing gene-wise percentage of pathogenic and VUS mutation. X-axis showing name of genes used in the study and Y-axis showing percentage of pathogenic (red) and VUS (blue) mutation.

From total 42 pathogenic mutations, 30 (71.42%) mutations were detected in BRCA1 gene, seven (16.66%) mutations were detected in BRCA2 gene, two (4.76%) mutations were detected in TP53 gene and one (2.38%) mutation each was detected in PALB2, BRIP1, and ATM genes respectively **Figures 4A, B**. The details of the mutations with clinical information of patients have been summarized in the **Table 1**. One pathogenic mutation c.5137+1G>A in BRCA1 gene was found to be present in 7/144 cases. Other two pathogenic mutations, which are c.5098delC and c.68_69delAG in BRCA1 gene, were found to be present in 4/144 and 3/144 cases respectively. Moreover, five pathogenic mutations, which are, c.1016delA in BRCA1 gene, c.1907C>G in BRCA2 gene, c.3331C>T in BRCA1 gene, c.5566C>T in BRCA1 gene and c.682-2A>G in BRCA2 gene were found to be present in 2/144 cases. Whereas, other pathogenic mutations were found to be present in 1/144 cases, **Table 1**. While in case of 18 cases from total six different families, pathogenic mutation was detected in five families. Three relatives of a cancer patient (P24) were found to have the same pathogenic mutation in in BRCA1 (c.5098delC) gene as the patient, and thus they might show symptoms of HBOC in future. None of the four relatives of other cancer patients (P8) were found to have any HBOC related mutations suggesting that they did not inherit the mutations. While from two sisters, P4 and P5, pathogenic mutation was only detected in BRCA1 (c.1016delA) in patient P5. Moreover, in the screening of the other two sisters P41 and P42, the same pathogenic mutation in BRCA2 (c.1907C>G) was identified. Further in two relatives of the patient P100, pathogenic mutation was detected in P136 relative in BRCA1 (c.5137+1G>A) gene, the same as patient P100.

**Table 1:** Showing the details of ClinVar pathogenic mutations and clinical details of patients in the study.

Pathogenic Mutations									
Patient ID	Age	Cancer Type	Hormone Receptor Status			Gene	Coding	Protein	Type
P59	36	Ca Breast Cancer	ER+	PR+	HER2-	TP53	c.1009C>T	p.Arg337Cys	Missense
P5	55	CA LT. Breast	ER-	PR-	HER2+	BRCA1	c.1016delA	p.Lys339fs	Frameshift Deletion
P11	14	LT CA. Ovary	NA	NA	NA	BRCA1	c.1016delA	p.Lys339fs	Frameshift Deletion
P135	36	CA RT. Breast	ER-	PR-	HER2-	BRCA1	c.1513A>T	p.Lys505Ter	Nonsense
P8	60	CA RT. Breast	ER-	PR-	HER2+	BRCA2	c.1773_1776 delTTAT	p.Ile591fs	Frameshift Deletion
P41	40	Ca Breast Cancer	NA	NA	NA	BRCA2	c.1907C>G	p.Ser636Ter	Nonsense
P42	54	B/L CA Ovary	NA	NA	NA	BRCA2	c.1907C>G	p.Ser636Ter	Nonsense
P29	38	CA RT. Breast	ER-	PR-	HER2-	BRCA1	c.220C>T	p.Gln74Ter	Nonsense
P85	33	Ca Breast Cancer	ER+	PR+	HER2-	PALB2	c.2488delG	p.Glu830fs	Frameshift Deletion
P101	56	Ca Breast Cancer	ER-	PR-	HER2-	BRCA1	c.2981_2982 delGT	p.Cys994Ter	Nonsense
P90	35	LT CA. Ovary	NA	NA	NA	BRCA1	c.3331_3334 delCAAG	p.Gln1111fs	Frameshift Deletion
P20	50	B/L CA Ovary	NA	NA	NA	BRCA1	c.3331C>T	p.Gln1111Ter	Nonsense
P116	48	LT CA. Ovary	NA	NA	NA	BRCA1	c.3331C>T	p.Gln1111Ter	Nonsense
P110	42	Ca Breast Cancer	ER-	PR-	HER2-	BRCA1	c.4120_4121 delAG	p.Ser1374Ter	Nonsense
P36	56	LT CA. Ovary	NA	NA	NA	BRCA1	c.4186C>A	p.Gln1396Lys	Missense
P125	42	B/L CA Ovary	NA	NA	NA	BRCA1	c.4548-1G>A	p.?	Unknown
P24	33	CA LT. Breast	ER+	PR+	HER2+	BRCA1	c.5098delC	p.Leu1700Ter	Nonsense
P127	36	BRCA1/2 Carrier	NA	NA	NA	BRCA1	c.5098delC	p.Leu1700Ter	Nonsense
P128	28	BRCA1/2 Carrier	NA	NA	NA	BRCA1	c.5098delC	p.Leu1700Ter	Nonsense
P129	38	BRCA1/2 Carrier	NA	NA	NA	BRCA1	c.5098delC	p.Leu1700Ter	Nonsense
P3	60	B/L CA Ovary	NA	NA	NA	BRCA1	c.5137+1G>A	p.?	Unknown
P18	45	RT CA. Ovary	NA	NA	NA	BRCA1	c.5137+1G>A	p.?	Unknown
P62	46	CA LT. Breast	NA	NA	NA	BRCA1	c.5137+1G>A	p.?	Unknown
P71	51	Ca Lt. Breast and Cervical Cancer	ER+	PR-	NA	BRCA1	c.5137+1G>A	p.?	Unknown
P100	57	Ca Breast Cancer	ER-	PR-	HER2-	BRCA1	c.5137+1G>A	p.?	Unknown
P136	55	Ca Breast Cancer	ER-	PR-	HER2-	BRCA1	c.5137+1G>A	p.?	Unknown
P123	35	CA LT. Breast	ER-	PR-	HER2-	BRCA1	c.5137+1G>A	p.?	Unknown
P53	21	CA RT. Breast	ER-	PR-	HER2+	BRCA1	c.5138-1G>A	p.?	Unknown
P111	32	B/L CA Ovary	NA	NA	NA	BRCA1	c.5158C>T	p.Arg1720Trp	Missense
P57	29	CA RT. Breast	NA	NA	NA	BRCA1	c.5566C>T	p.Arg1856Ter	Nonsense
P27	41	CA LT. Breast	ER-	PR-	HER2-	BRCA1	c.5566C>T	p.Arg1856Ter	Nonsense
						ATM	c.8432delA	p.Lys2811fs	Frameshift Deletion
P30	36	B/L CA Ovary & CA Breast	NA	NA	NA	BRCA1	c.5572T>C	p.Trp1858Arg	Missense
P52	37	CA LT. Breast	ER-	PR-	HER2+	BRCA2	c.6450delA	p.Val2151fs	Frameshift Deletion
P94	46	CA LT. Breast	NA	NA	NA	BRCA1	c.68_69delAG	p.Glu23fs	Frameshift Deletion
P99	38	Ca Breast Cancer	ER-	PR-	HER2-	BRCA1	c.68_69delAG	p.Glu23fs	Frameshift Deletion
P47	37	CA RT. Breast	NA	NA	NA	BRCA1	c.68_69delAG	p.Glu23fs	Frameshift Deletion
						BRIP1	c.2992_2993 delAA	p.Lys998fs	Frameshift Deletion
P1	37	CA RT. Breast	ER+	PR+	HER2+	BRCA2	c.682-2A>G	p.?	Unknown
P40	62	Ca Breast Cancer	ER+	PR+	HER2+	BRCA2	c.682-2A>G	p.?	Unknown
P87	48	CA RT. Breast	ER-	PR-	HER2+	TP53	c.733G>A	p.Gly245Ser	Missense
P117	47	CA RT. Breast	ER-	PR-	HER2-	BRCA2	c.956_957insA	p.Asn319fs	Frameshift Insertion

#### VUS

**Figure 5:**
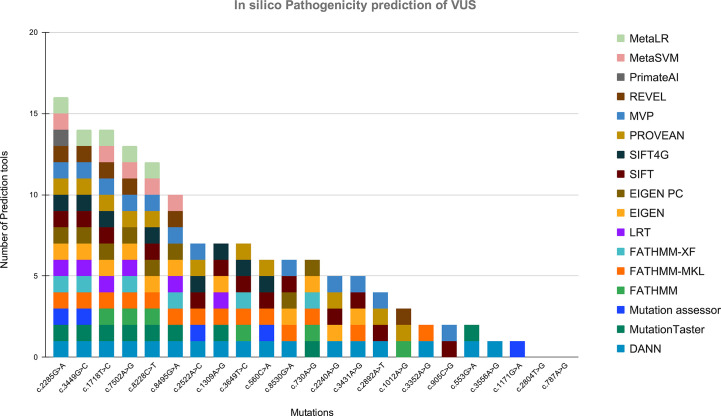
Showing the *in silico* pathogenicity prediction of VUS. X-axis shows the mutation found in our study, and Y-axis shows the number of tools predicting the variant as a pathogenic mutation.

In this study, we also detected 25 missense variants **Table 2** that are clinically placed in the category of “variants of uncertain significance-VUS” by ClinVar (20). Clinical significance of these VUS was evaluated by Varsome which subjects each mutation to 17 *in silico* pathogenicity prediction tools including Mutation Taster^®^, Mutation Assessor, FATHMM, FATHMM-MKL, FATHMM-XF, LRT, Eigen, Eigen PC, SIFT, SIFT 4G, PROVEAN, MVP, REVEL, PrimateAI, MetaSVM, MetalR, and DANN. Varsome is the human genome variant search engine for prediction of pathogenicity by multiple tools (17). Out of these 25 VUS, c.2285G>A in BRIP1 was predicted to be pathogenic by 16 of the 17 pathogenicity prediction tools, c.3449G>C in ATM and c.1718T>C in BARD1 were predicted to be pathogenic by 14 of the 17 pathogenicity prediction tools, c.7502A>G in ATM was predicted to be pathogenic by 13 of the 17 tools, while c.8228C>T in ATM was predicted to be pathogenic by 12 out of the 17 tools, c.8495G>A and c.2522A>C in ATM was predicted to be pathogenic by 10 and 7 tools respectively **Figure 5**.

**Table 2:** Showing the details of Variants of Uncertain Significance (VUS) found in the study.

VUS						
Sr. No.	Gene	Chromosomal Location	Coding	Protein	Type	Pathogenicity prediction (out of 17 tools)
1	BRIP1	chr17:59820468	c.2285G>A	p.Arg762His	Missense	16
2	ATM	chr11:108151768	c.3449G>C	p.Arg1150Thr	Missense	14
3	BARD1	chr2:215610538	c.1718T>C	p.Ile573Thr	Missense	14
4	ATM	chr11:108201135	c.7502A>G	p.Asn2501Ser	Missense	13
5	ATM	chr11:108206648	c.8228C>T	p.Thr2743Met	Missense	12
6	ATM	chr11:108216546	c.8495G>A	p.Arg2832His	Missense	10
7	ATM	chr11:108137953	c.2522A>C	p.Asp841Ala	Missense	7
8	BARD1	chr2:215645289	c.1309A>G	p.Ile437Val	Missense	7
9	BRCA1	chr17:41243899	c.3649T>C	p.Ser1217Pro	Missense	7
10	BRCA2	chr13:32945135	c.8530G>A	p.Glu2844Lys	Missense	6
11	RAD51C	chr17:56787244	c.730A>G	p.Ile244Val	Missense	6
12	BRCA2	chr13:32910732	c.2240A>G	p.Glu747Gly	Missense	5
13	BRIP1	chr17:59760976	c.3431A>G	p.Glu1144Gly	Missense	5

### Comparison of Mutational Profile of Indian HBOC Patients With Other Populations

When the pathogenic mutations and VUS found in our data were compared with the GnomeAD database, we found that mutational frequency distribution differs in Indian patients from other populations. On comparing the minor allele frequencies of clinically significant mutations, including pathogenic and VUS from Indian HBOC patients with that of other populations such as South Asian, East Asian, Latino, Ashkenazi, European-Finnish, European-non-Finnish and African populations, a separation of mutational frequencies of Indian cohort with other global population was observed, suggesting a different mutational frequency profile of Indian HBOC patients. We first compared the minor allele frequencies of pathogenic mutations detected in our study with that of other populations mentioned in the additional file (see **Supplementary Table 1**). Secondly, we compared the minor allele frequencies of variants of uncertain significance (VUS) detected in our study with that of other populations mentioned in the additional file (see **Supplementary Table 2**). It was found that 11 out of 25 VUS detected in our study namely, c.3431A>G in BRIP1, c.553G>A in BRIP1, c.3556A>G in BRIP1, c.787A>G in TP53, c.8530G>A in BRCA2, c.2240A>G in BRCA2, c.2892A>T in BRCA2, c.905C>G in BRCA2, c.3449G>C in ATM, c.2522A>C in ATM, and c.3352A>G in ATM were only detected in South Asian population out of all the populations considered in the comparison study.

## Discussion

### Population Specific Scenario in Genetic Predisposition

In the present, study Amplicon sequencing of 14 potential high risk genes was performed to uncover mutational profiles of 144 Indian patients having a family history of breast and or ovarian cancer.

#### Distribution of Mutations in Non-BRCA Genes

The study indicates that in the Indian population, mutations in the BRCA1/2 genes are the major contributors 37/144 (25.69%) for hereditary breast and/or ovarian cancers. Whereas the non-BRCA genes contribute 5/144 (3.47%) for hereditary breast and/or ovarian cancers; two mutations were detected in TP53 gene, and one mutation each was detected in PALB2, BRIP1, and ATM genes respectively. The contribution of pathogenic mutations by non-BRCA genes is 3.47% which is lower as compared to the other Indian studies involving multi-gene panel which might be due to lower number of cases in our study (12). Multigene panels ranging from 13 genes to 29 genes have been used in different studies for hereditary breast and ovarian cancer in different population and pathogenic variants in other non-BRCA genes have been consistently reported (21, 22)

#### Clinically Significant Mutations in Indian Patients

In our study, two recurrent BRCA1 pathogenic mutations were observed in more than three cases, and these mutations are c.5137+1G>As and c.5098delC (12, 23–25), out of which the most frequent pathogenic mutation c.5137+1G>A has also been reported in another Indian study (12). While, c.5098delC, has not been reported in any other Indian study as per our best knowledge. It was also found that a pathogenic mutation, c.5137+1G>A in BRCA1, is found nowhere in the other populations considered for comparison except in South Asian population (MAF: 3.27E-05), suggesting it to be a possible biomarker for HBOC in Indian population. Other pathogenic mutations which were detected in 2/144 cases each (MAF:1.39E-02) in our study are c.3331C>T and c.5566C>T in BRCA1, out of which, c.3331C>T is found to be detected only in East Asian population (MAF: 5.44E-05), while c.5566C>T is found to be detected only in Latino population (MAF: 2.89E-05) and South Asian population (MAF: 6.53E05). Another pathogenic mutation, c.4548-1G>A in BRCA1 (MAF: 6.94E-03) is found to be detected in only the South Asian population (MAF: 9.80E-05). The findings obtained suggest that the recurrent pathogenic mutations that are detected in our study and are not found to be detected in any other population except the South Asian population can be possible biomarkers for HBOC in the Indian population. Other recurrent mutations detected in our study such as c.5098delC in BRCA1, c.682-2A>G in BRCA2, c.1907C>G in BRCA2 and c.1016delA in BRCA1 were not available in the GnomAD database for comparison of their frequencies in different populations. Another pathogenic mutation, c.68_69delAG in BRCA1 (MAF: 2.08E-02) is also found to be highly frequent in Ashkenazi population (MAF: 4.05E-03) and is also reported as a founder mutation in the Ashkenazi Jewish population suggesting that the Ashkenazi founder mutation is not only present in the Ashkenazi descent but also present in non-Ashkenazi descent in Israel, Spain, Poland, India, and other countries in Central and Eastern Europe (26–29). The frequency range of this mutation in the Indian population is reported to be 0.5–4.1% (27). The frequency of this mutation in the present study cohort was 2.08% (3/144), which falls under the reported range of 0.5–4.1%. Also, 11 VUS coincide only with the South Asian population and are also predicted to be disease-causing by prediction tools; it suggests that these VUS could be possible biomarkers for HBOC in the Indian population. Observation of differences in mutational spectrum of Indian cohort and other global populations supports the need of generating population specific mutational profiles, which will help in designing more accurate, effective strategies in HBOC risk assessment/screening, diagnosis and therapy.

### Important Genes for Indian HBOC Patients

Clinically important genes have been found to have mutations in many genes from the 14 genes included in this study. We found pathogenic mutations in six genes including BRCA1, BRCA2, BRIP1, TP53, ATM, and PALB2. We also found VUS predicted to be pathogenic in six genes including BRCA1, BRCA2, BRIP1, ATM, RAD51C, and BARD1. Hence, it is suggested that apart from the BRCA genes, other non-BRCA genes such as ATM, PALB2, and TP53 also contribute to HBOC in the Indian population. The findings of the current study strongly emphasize incorporation of multi-gene panels, including high-penetrance genes such as BRCA1/2 and moderate-risk or non-BRCA genes in genetic testing of Indian HBOC patients. These findings will help in better understanding of the contribution of high-risks and moderate-risks genes in onset, prevention, and management of hereditary breast and ovarian cancer in Indian patients. The above findings can be further validated on much wider populations for developing future risk assessment and screening programs of HBOC in India.

## Conclusions

Mutational frequency patterns of Indian hereditary breast and ovarian cancer patients are different from the other global populations. High frequency clinically significant pathogenic mutations, recurrent novel mutations, and variants of uncertain significance, which were predicted to be disease-causing by majority of *in silico* tools, are suggested to be biomarkers for screening and risk assessment strategies of Indian HBOC patients. Five variants of uncertain significance that were predicted to be pathogenic or disease-causing by more than 10 of 17 pathogenicity prediction tools can be further studied and validated on more numbers of samples to understand their role in HBOC. Further validation of these mutations on more number of Indian HBOC patients shall strengthen their role and associations with disease development and progression, which in turn will also help in designing strategies in the prevention, diagnosis, and better disease management.

## Data Availability Statement

We have deposited our sequence data in NCBI and our submission Bioproject Accession number is PRJNA675417.

## Ethics Statement

The studies involving human participants were reviewed and approved by Institutional Review Board, Gujarat Cancer Research Institute Registration no.: ECR/41/Inst/GJ/2013/RR-16, date of registration: 16th April 2016. The patients/participants provided their written informed consent to participate in this study.

## Author Contributions

CJ, MJ, PB, and IS were responsible for the conception and design. FS, NB, BT, IS, MK, and KP were responsible for the acquisition of samples and filling out of the consent forms. MK, KP, and MS were involved in interpretation of the data. MK and KP contributed to the manuscript writing. KS was responsible for the genetic counseling of patients. All authors contributed to the article and approved the submitted version.

## Funding

Gujarat Biotechnology Research Centre (GBRC) and Gujarat Cancer Research Institute (GCRI) were funded by Gujarat State Biotechnology Mission (GSBTM), Department of Science and Technology, Government of Gujarat (21WU41).

## Conflict of Interest

The authors declare that the research was conducted in the absence of any commercial or financial relationships that could be construed as a potential conflict of interest.

## Abbreviations

ATM, ataxia-telangiectasia mutated; BARD1, BRCA1 Associated RING Domain 1; BLAST, Basic Local Alignment Search Tool; BOC, Breast and Ovarian Cancer; BRCA1, Breast cancer type 1 susceptibility protein; BRCA2, Breast cancer type 2 susceptibility protein; BRIP1, BRCA1 interacting protein 1 gene; CHEK2, checkpoint kinase 2 gene; ERBB2, Erb-B2 Receptor Tyrosine Kinase 2; HBOC, Hereditary Breast and Ovarian Cancer; MAF, Minor Allele Frequency; MVP, Missense Variant Pathogenicity; NBN, Nibrin; NCCN, National Comprehensive Cancer Network; NGS, Next Generation Sequencing; PALB2, Partner And Localizer Of BRCA2; PTEN, Phosphatase And Tensin Homolog; RAD51C, RAD51 Paralog C; STK11 Serine/Threonine Kinase 11; TP53, Tumor Protein P53; VUS, Variants of Uncertain Significance.
