# Supplementary material for: Mutational Landscape for Indian Hereditary Breast and Ovarian Cancer Cohort Suggests Need for Identifying Population Specific Genes and Biomarkers for Screening
Source: Front Oncol. 2021 Jan 21;10:568786. doi: 10.3389/fonc.2020.568786 (PMC7859489; doi:10.3389/fonc.2020.568786)
Supplement: Supplementary file 1 [file Table_1.docx]

**Supplementary Table 1:**

Comparison of minor allele frequencies of pathogenic mutations detected in our study with that of other populations.

| **Details** | | **Allele Frequency (gnomAD)** | | | | | | |
| --- | --- | --- | --- | --- | --- | --- | --- | --- |
| **Location** | **coding** | **South Asian** | **European (Finnish)** | **Latino** | **Ashkenazi Jewish** | **East Asian** | **European (Non-Finnish)** | **African** |
| chr17:41219624 | c.5137+1G>A | 3.27E-05 | 0.00E+00 | 0.00E+00 | 0.00E+00 | 0.00E+00 | 0.00E+00 | 0.00E+00 |
| chr17:41244217 | c.3331C>T | 0.00E+00 | 0.00E+00 | 0.00E+00 | 0.00E+00 | 5.44E-05 | 0.00E+00 | 0.00E+00 |
| chr17:41197784 | c.5566C>T | 6.53E-05 | 0.00E+00 | 2.89E-05 | 0.00E+00 | 0.00E+00 | 0.00E+00 | 0.00E+00 |
| chr17:7577548 | c.733G>A | 0.00E+00 | 0.00E+00 | 0.00E+00 | 0.00E+00 | 0.00E+00 | 0.00E+00 | 4.01E-05 |
| chr16:23640986 | c.2488delG | 3.27E-05 | 0.00E+00 | 0.00E+00 | 0.00E+00 | 0.00E+00 | 0.00E+00 | 0.00E+00 |
| chr17:41276044 | c.68_69delAG | 1.31E-04 | 0.00E+00 | 2.82E-05 | 4.05E-03 | 0.00E+00 | 8.54E-05 | 0.00E+00 |
| chr17:41215948 | c.5158C>T | 0.00E+00 | 9.24E-05 | 0.00E+00 | 0.00E+00 | 5.44E-05 | 1.76E-05 | 0.00E+00 |
| chr13:32906565 | c.956_957insA | 0.00E+00 | 0.00E+00 | 0.00E+00 | 0.00E+00 | 5.87E-05 | 9.28E-05 | 0.00E+00 |
| chr17:41226539 | c.4548-1G>A | 9.80E-05 | 0.00E+00 | 0.00E+00 | 0.00E+00 | 0.00E+00 | 0.00E+00 | 0.00E+00 |
| chr17:59761413 | c.2992_2993delAA | 0.00E+00 | 0.00E+00 | 0.00E+00 | 0.00E+00 | 0.00E+00 | 0.00E+00 | 6.16E-05 |
| chr17:7574018 | c.1009C>T | 0.00E+00 | 0.00E+00 | 0.00E+00 | 0.00E+00 | 0.00E+00 | 0.00E+00 | 0.00E+00 |
| chr17:41234592 | 4186C>A | 0.00E+00 | 0.00E+00 | 0.00E+00 | 0.00E+00 | 0.00E+00 | 0.00E+00 | 1.15E-04 |

**Supplementary Table 2:**

Comparison of minor allele frequencies of variants of uncertain significance (VUS) detected in our study with that of other populations.

| **Details** | | **Allele Frequency (gnomAD)** | | | | | | |
| --- | --- | --- | --- | --- | --- | --- | --- | --- |
| **Location** | **coding** | **South Asian** | **European (Finnish)** | **Latino** | **Ashkenazi Jewish** | **East Asian** | **European (Non-Finnish)** | **African** |
| chr2:215610538 | 1718T>C | 0.00E+00 | 0.00E+00 | 2.82E-05 | 0.00E+00 | 0.00E+00 | 4.65E-05 | 0.00E+00 |
| chr2:215645289 | 1309A>G | 0.00E+00 | 4.69E-05 | 0.00E+00 | 0.00E+00 | 0.00E+00 | 0.00E+00 | 0.00E+00 |
| chr17:59760976 | 3431A>G | 5.56E-04 | 0.00E+00 | 0.00E+00 | 0.00E+00 | 0.00E+00 | 0.00E+00 | 0.00E+00 |
| chr17:59924536 | 553G>A | 1.05E-03 | 0.00E+00 | 0.00E+00 | 0.00E+00 | 0.00E+00 | 0.00E+00 | 0.00E+00 |
| chr17:59760851 | 3556A>G | 9.80E-05 | 0.00E+00 | 0.00E+00 | 0.00E+00 | 0.00E+00 | 0.00E+00 | 0.00E+00 |
| chr17:7577151 | 787A>G | 8.29E-04 | 0.00E+00 | 0.00E+00 | 0.00E+00 | 0.00E+00 | 0.00E+00 | 0.00E+00 |
| chr13:32945135 | 8530G>A | 4.57E-04 | 0.00E+00 | 0.00E+00 | 0.00E+00 | 0.00E+00 | 0.00E+00 | 0.00E+00 |
| chr13:32910732 | 2240A>G | 2.94E-04 | 0.00E+00 | 0.00E+00 | 0.00E+00 | 0.00E+00 | 0.00E+00 | 0.00E+00 |
| chr13:32911384 | 2892A>T | 3.70E-04 | 0.00E+00 | 0.00E+00 | 0.00E+00 | 0.00E+00 | 0.00E+00 | 0.00E+00 |
| chr13:32906520 | 905C>G | 2.23E-04 | 0.00E+00 | 0.00E+00 | 0.00E+00 | 0.00E+00 | 0.00E+00 | 0.00E+00 |
| chr11:108151768 | 3449G>C | 1.47E-03 | 0.00E+00 | 0.00E+00 | 0.00E+00 | 0.00E+00 | 0.00E+00 | 0.00E+00 |
| chr11:108137953 | 2522A>C | 1.37E-03 | 0.00E+00 | 0.00E+00 | 0.00E+00 | 0.00E+00 | 0.00E+00 | 0.00E+00 |
| chr11:108150285 | 3352A>G | 1.63E-03 | 0.00E+00 | 0.00E+00 | 0.00E+00 | 0.00E+00 | 0.00E+00 | 0.00E+00 |
| chr17:59763298 | 2804T>G | 1.44E-03 | 0.00E+00 | 0.00E+00 | 0.00E+00 | 0.00E+00 | 2.64E-05 | 0.00E+00 |
| chr16:68847249 | 1171G>A | 9.80E-05 | 7.96E-05 | 0.00E+00 | 0.00E+00 | 0.00E+00 | 0.00E+00 | 0.00E+00 |
| chr17:56787244 | 730A>G | 2.29E-04 | 0.00E+00 | 0.00E+00 | 9.92E-05 | 0.00E+00 | 3.52E-05 | 0.00E+00 |
| chr11:108201135 | 7502A>G | 1.24E-03 | 0.00E+00 | 8.47E-05 | 0.00E+00 | 0.00E+00 | 7.76E-06 | 0.00E+00 |
| chr11:108206648 | 8228C>T | 0.00E+00 | 0.00E+00 | 2.82E-05 | 0.00E+00 | 5.02E-05 | 2.33E-05 | 0.00E+00 |
| chr17:59820468 | 2285G>A | 1.96E-04 | 0.00E+00 | 2.89E-05 | 0.00E+00 | 1.09E-04 | 6.16E-05 | 6.15E-05 |
| chr17:41243899 | 3649T>C | 1.96E-04 | 4.78E-04 | 2.82E-05 | 0.00E+00 | 2.01E-04 | 7.75E-06 | 0.00E+00 |
| chr11:108216546 | 8495G>A | 4.90E-04 | 0.00E+00 | 2.82E-05 | 0.00E+00 | 2.01E-04 | 7.75E-05 | 4.01E-05 |
